# Supplementary material for: Terahertz‐Nanoscale Visualization of the Microscopic Spin‐Charge Architecture of Colossal Magnetoresistive Switching
Source: Adv Sci (Weinh). 2026 Jul 24;13(59):e76799. doi: 10.1002/advs.76799 (PMC13398138; doi:10.1002/advs.76799)
Supplement: Supplementary file 1 [file ADVS-13-e76799-s001.pdf]

Supplementary Information for Terahertz-nanoscale visualization of the microscopic spin-charge architecture of colossal magnetoresistive switching

Samuel Haeuser[1,2], Randall K. Chan[1,2], Richard H. J. Kim[2],
Joong-Mok Park[2], Martin Mootz[2], Thomas Koschny[2], Jigang Wang[1,2†]

[1] *Department of Physics and Astronomy,*
Iowa State University, Ames, Iowa 50011, USA.

[2]*Ames National Laboratory of U.S. Department of Energy, Ames, Iowa 50011, USA.*

[†]*Corresponding author: jgwang@iastate.edu*

(Dated: July 13, 2026)

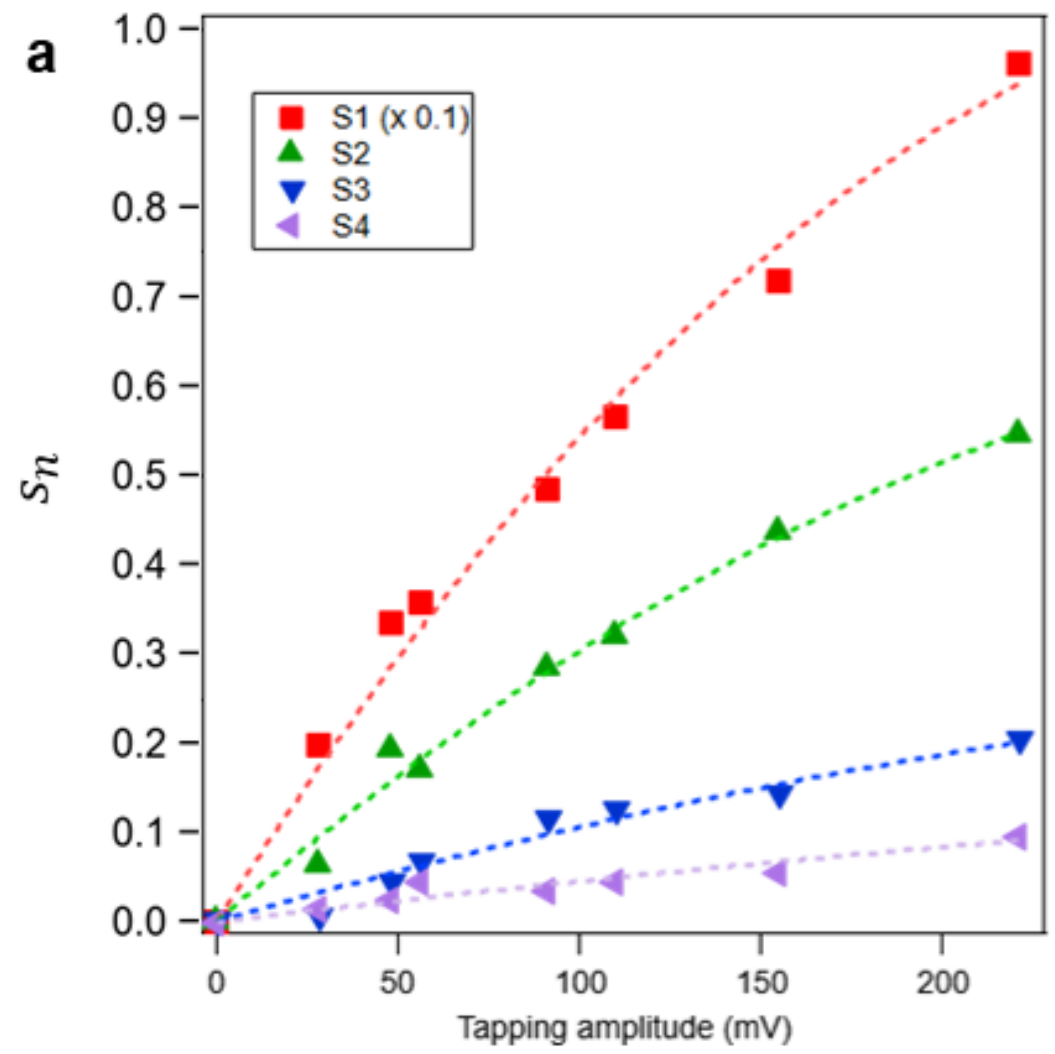

Figure S1. **Near-field signal calibration and tapping amplitude dependence. a)** THz near-field signal magnitudes S_n ($n = 1, 2, 3, 4$) as a function of tapping amplitude recorded at 29 K under zero-field conditions. These measurements provide a crucial calibration reference to compensate for magnetic-field-induced fluctuations in the cantilever mechanical response.

I. DATA ANALYSIS

In previous works we investigated the effect of the magnetic field on the solid platinum probe used for THz SNOM measurements[1]. We previously concluded that the AFM performance remains unchanged but the tapping amplitude is reduced, thus increasing the noise level of the system. To accurately calibrate the THz near field signal size under different magnetic field we measure the zero-field THz near-field response under many different tapping amplitudes at 29 K, as shown in Fig. S1a. We then use this as a calibration reference for the measured tapping amplitude measured at different fields. The calibrated near-field response is plot in Figure 2a.

II. CONTROLLED EXPERIMENT

To validate the sensitivity of our cm-THz-sSNOM and characterize the near-field contrast between dielectric and conductive phases, we benchmarked the field-driven transition against

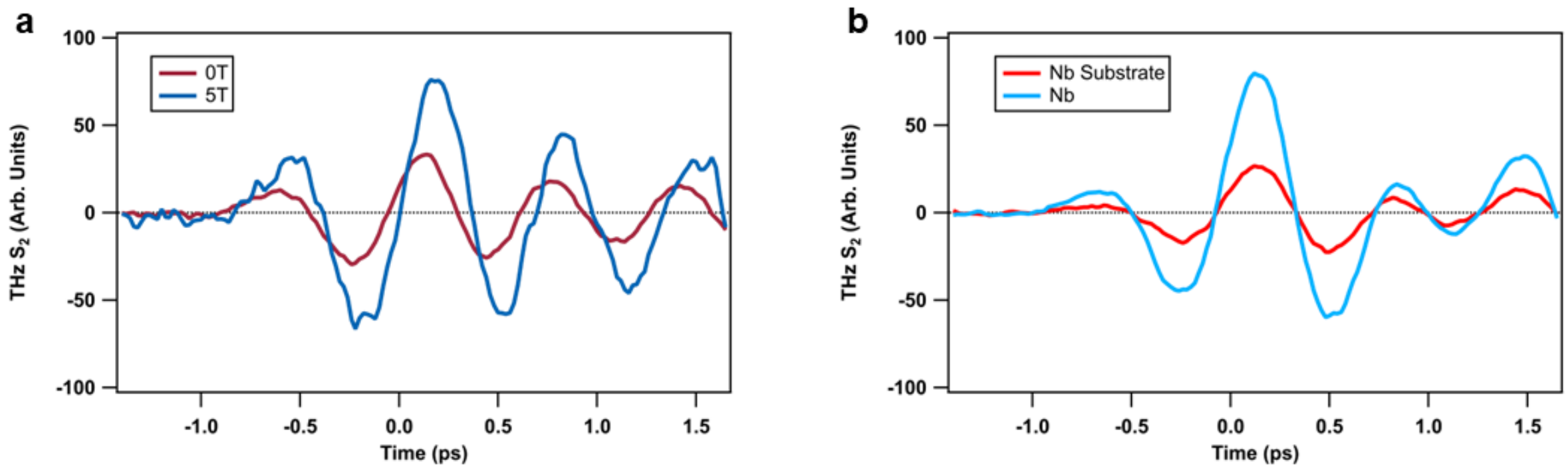

Figure S2. **Benchmarking THz near-field contrast against metallic control samples. a)** THz near-field s_2 time-domain waveforms for the PCMO single crystal at 29 K in the charge-ordered insulating state (0 T, red) and the field-induced metallic state (5 T, blue). **b)** Comparative THz s_2 time-domain data for a 150–170 nm thick Nb thin film (blue) and its bare sapphire substrate (red) at room temperature. The experimental contrast ratio of 2.28 observed during the PCMO transition is quantitatively benchmarked against the 2.99 ratio of the Nb-to-substrate system, confirming the high sensitivity of the cm-THz-sSNOM to field-driven electronic switching.

a metallic thin-film standard. Figure S2a presents the THz time-domain near-field waveforms for the PCMO sample at 0 T (charge-ordered insulating state) and 5 T (ferromagnetic metallic state). For comparison, Figure S2b shows the time-domain response for a 150–170 nm thick Nb thin film relative to its bare sapphire substrate at room temperature. Our benchmark measurements reveal a signal contrast ratio of 2.99 for the Nb-to-substrate system, while the PCMO insulator-to-metal transition (IMT) yields a contrast of 2.28. This slight variance in contrast magnitude is primarily attributed to the inherent differences in the complex conductivity and dielectric permittivity between the sapphire substrate and the 0 T insulating phase of $Pr0.67Ca0.33MnO_3$.

III. ELLIPSOIDAL DIPOLE MODEL

We simulate the probe–sample interaction using an ellipsoidal dipole model positioned above a conducting surface. Figure S3a illustrates the calculated electrostatic potential of the ellipsoid in contact with the surface, along with the corresponding lateral profile of the electric field (E-field) at the surface interface ($z = 0$). Given that the scattered near-field

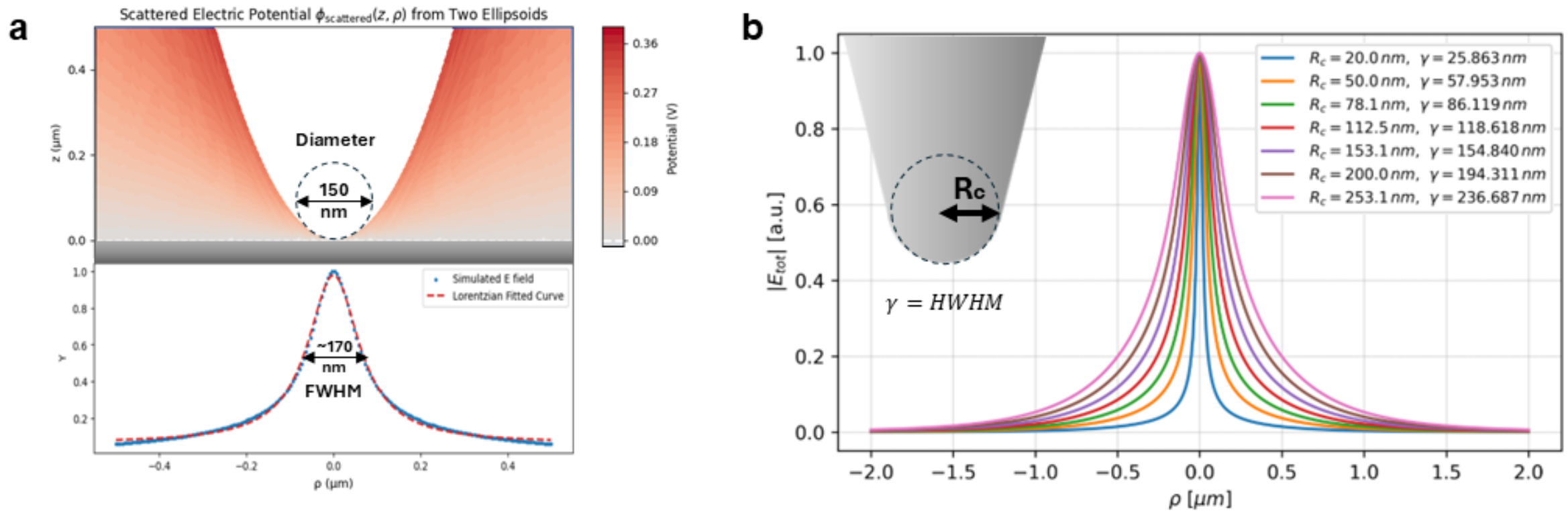

Figure S3. **Electrostatic modeling of the probe-sample interaction and near-field confinement. a) (Top)** Simulated electrostatic potential for the ellipsoidal dipole model in contact with a conducting surface. **(Bottom)** Corresponding lateral profile (line-cut) of the electric field (E-field) at the surface interface ($z = 0$), where the dashed line represents a Lorentzian fit with a full-width at half-maximum (FWHM) of ∼170 nm. **b)** Calculated E-field line-cuts at the surface ($z = 0$) for varied probe curvature radii (R_c), where γ denotes the half-width at half-maximum (HWHM) of the resulting Lorentzian distribution. The consistent Lorentzian behavior across a broad range of probe geometries demonstrates the robustness of the near-field interaction model.

signal is proportional to the local E-field, the signal amplitude A for a real dielectric system is expected to scale as αA, where $\alpha = (\epsilon_r - 1)/(\epsilon_r + 1)$ represents the dielectric response of the surface [2, 3]. In the limit of high permittivity ($|\epsilon_r| \gg 1$), the system approaches the behavior of a perfect conductor. Figure S3b presents the calculated line-cuts of the E-field at $z = 0$ for probes of varying curvature radii (R_c), where γ denotes the half-width at half-maximum (HWHM) of a fitted Lorentzian profile. We observe that the near-field distribution consistently follows a Lorentzian profile across a broad range of probe geometries, indicating that this interaction remains robust regardless of specific tip apex dimensions.

IV. CONVOLUTION BLUR MODEL

To create a convolution kernel to achieve the results from the ellipsoidal model, we simply can define a 2D Lorentzian function and truncate the tails to a reasonable size based on the FWHM (typically 4 × FWHM) to save time in computing.

$$L[i,j] = \frac{(\frac{1}{2}\Gamma)^2}{((i-r)^2 + (j-r)^2 + (\frac{1}{2}\Gamma)^2)}$$

Where Γ is the FWHM and r is distance the center of Lorentzian to the truncated edge. When populating a kernel matrix to use practically for a convolution it is common to define a useful size based on the truncated parameters by defining $r = T * \Gamma/2$ where T is the truncation parameter and $L = numpy.zeros(shape = (2r, 2r))$.

In real samples with the magnetic induced conductivity changes, the atomic scale domains are much smaller than the probe. The simulated domains are populated using a simple random generator function through pythons Numpy package (*numpy.random.choice*) that outputs 1 or 0 for each entry in an array based on the overall filling factor extracted from the experimental data: representing the conducting or non-conducting atomically bound spin flips. The Lorentzian kernel was then scaled to the corresponding pixel to the parameters of the FWHM distribution of the elliptical model to apply the appropriate distribution of blur. Finally, the Lorentzian kernel is convoluted with the simulated domains. To match the experimental data, the model image is further processed to "pixelate" it by down-sampling this to the same experimental nm/pixel size used for scanning (33 nm/pixel).

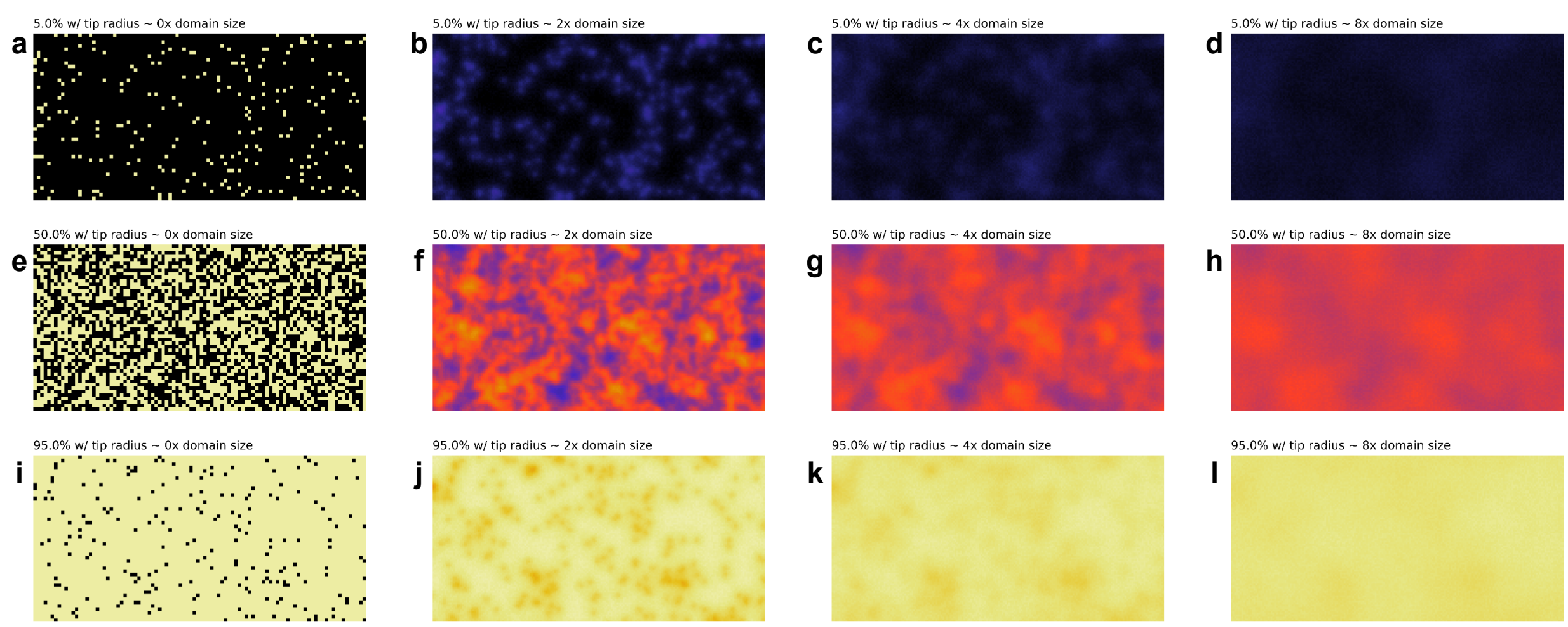

Figure S4. **Randomly populated domains under a Lorentzian convolution a-d** Modeled domains for conducting volume fractions of 5.0% at unfiltered, 37.5 nm, 18.75 nm, 9.38 nm domain size. **e-f** Modeled domains for conducting volume fractions of 50.0% at unfiltered, 37.5 nm, 18.75 nm, 9.38 nm domain size. **i-l** Modeled domains for conducting volume fractions of 95.0% at unfiltered, 37.5 nm, 18.75 nm, 9.38 nm domain size.

To evaluate how robustly our model separates sub-resolution domain sizes, we conducted a systematic simulation series modeling near-field images across various conducting volume fractions (5.0%, 50.0%, and 95.0%) under a generalized Lorentzian convolution kernel, as presented in Fig. S4 a, e, i respectively .

Given our experimental tip radius of 75 nm, the columns from left to right in: 0x: The unfiltered, raw stochastic domain grid, Fig. S4 a, e, i; 1x: A domain size equal to the tip radius (37.5 nm, , Fig. S4 b, f, j) 2x: A domain size equal to half the tip radius (18.75 nm Fig. S4 c, g, k); 4x: A domain size equal to a quarter of the tip radius (9.38 nm, Fig. S4 d, h, l). By inspecting the intermediate state (50.0% filling fraction Fig. S4 e-h), a crucial trend emerges:

For domain sizes of 37.5 nm (2x) and 18.75 nm (4x), the convolved images exhibit highly pronounced, visible spatial patchiness and intense pixel-to-pixel fluctuations.

Even when the domain size is reduced to 9.38 nm (8x), the image retains a discernible spatial texture and intensity variation, even though the exact geometric shapes of individual domains are completely blurred out.

Because our experimental near-field maps (Fig. 3e–3j) remain strikingly homogeneous and lack this patchy contrast, any coalesced conducting regions must remain strictly below ∼15 nm prior to reaching the global percolation threshold. If the domains were larger than ∼15 nm, the tip-sample interaction volume would inevitably pick up these localized density fluctuations, manifesting as measurable real-space contrast variations that exceed our experimental noise floor.

V. COMPARATIVE GAUSSIAN CONVOLUTION ANALYSIS

To ensure the robustness of our spatial analysis, we performed a comparative study using a Gaussian-like blur convolution kernel to evaluate resolution limits under alternative tip-interaction assumptions. This approach follows the same population methodology described in the main text but utilizes a Gaussian distribution to define the near-field region of influence. Figures S5a–f display the same stochastic population of conducting and insulating domains utilized in the primary model, with the conducting volume fractions determined by experimental data. Figures S5g–l show the resulting Gaussian-convolved images, with the corresponding intensity distribution shown in Fig. S5m. While the Gaussian model yields

qualitatively similar results to the Lorentzian model, we find that the estimated domain sizes for individual magnetic fields are slightly smaller under the Gaussian assumption.

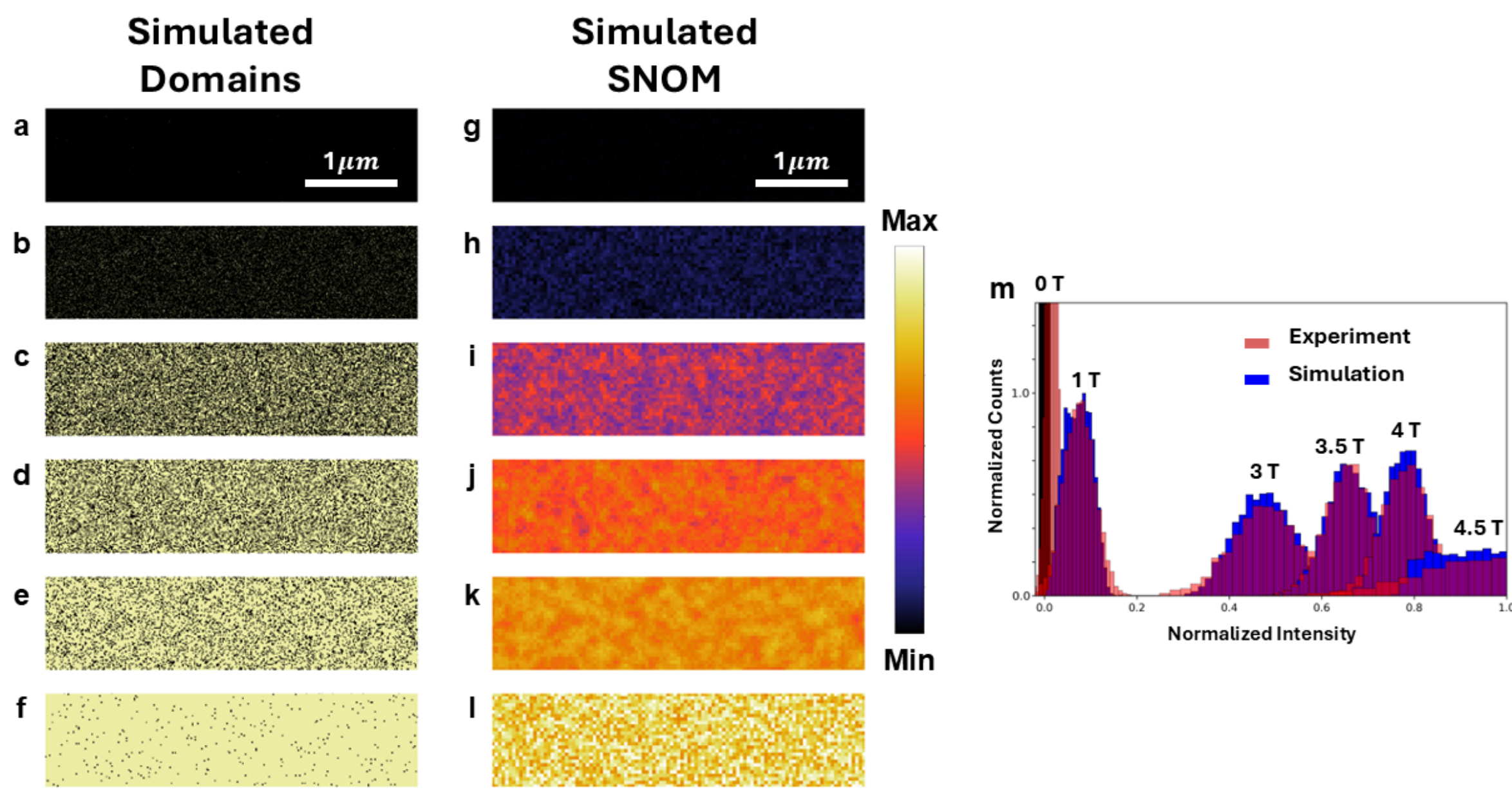

Figure S5. a-f Modeled individual spin flips for 0 T, 1 T, 3 T, 3.5 T, 4 T, 4.5 T respectively. g-l Modeled THz near field images for 0 T, 1 T, 3 T, 3.5 T, 4 T, 4.5 T, respectively, using the respective modeled spin flips in (a)-(d) under a global scale (4 μm x 1 μm) and gaussian convolution kernel. m Histogram of spatial distribution for experiment (red) and model (blue) for image data.

ACKNOWLEDGMENTS

Work at Ames National Laboratory was supported by the U.S. Department of Energy (DOE), Basic Energy Sciences, Division of Materials Sciences & Engineering, under Contract No. DE-AC02-07CH11358.

AUTHOR CONTRIBUTIONS

J.W. conceived and supervised the project. S.H., R.H.J.K., R.K.C., and J.M.K., performed the THz nano-imaging measurements. T.K., S.H., R.H.J.K., and M.M., developed the near-field model. S.H., and R.K.C., analyzed the data and performed simulations with discussions from all authors. The paper is written by S.H., and J.W., with input from all authors

COMPETING INTERESTS

The authors declare that they have no competing financial interests.

DATA AVAILABILITY STATEMENT

The data that support the plots within this paper and other findings of this study are available from the corresponding author upon reasonable request.

[1] S. Haeuser, R. H. Kim, J.-M. Park, R. K. Chan, M. Imran, T. Koschny, and J. Wang, Instruments **8**, 21 (2024).

[2] B. Knoll and F. Keilmann, Nature **399**, 134 (1999), URL `https://www.nature.com/articles/20154`.

[3] B. Knoll and F. Keilmann, Optics communications **182**, 321 (2000).
